# Polystyrene-Divinylbenzene-Based Adsorbents Reduce Endothelial Activation and Monocyte Adhesion Under Septic Conditions in a Pore Size-Dependent Manner

**DOI:** 10.1007/s10753-016-0408-1

**Published:** 2016-08-08

**Authors:** Tanja Eichhorn, Sabine Rauscher, Caroline Hammer, Marion Gröger, Michael B. Fischer, Viktoria Weber

**Affiliations:** 1 Christian Doppler Laboratory for Innovative Therapy Approaches in Sepsis, Department for Health Sciences and Biomedicine, Danube University Krems, Dr.-Karl-Dorrek-Strasse 30, 3500 Krems, Austria; 2 Core Facility Imaging, Medical University of Vienna, Vienna, Austria; 3 Department of Blood Group Serology and Transfusion Medicine, Medical University of Vienna, Vienna, Austria

**Keywords:** cytokine adsorption, endothelium, sepsis, lipopolysaccharide, monocyte adhesion

## Abstract

Endothelial activation with excessive recruitment and adhesion of immune cells plays a central role in the progression of sepsis. We established a microfluidic system to study the activation of human umbilical vein endothelial cells by conditioned medium containing plasma from lipopolysaccharide-stimulated whole blood or from septic blood and to investigate the effect of adsorption of inflammatory mediators on endothelial activation. Treatment of stimulated whole blood with polystyrene-divinylbenzene-based cytokine adsorbents (average pore sizes 15 or 30 nm) prior to passage over the endothelial layer resulted in significantly reduced endothelial cytokine and chemokine release, plasminogen activator inhibitor-1 secretion, adhesion molecule expression, and in diminished monocyte adhesion. Plasma samples from sepsis patients differed substantially in their potential to induce endothelial activation and monocyte adhesion despite their almost identical interleukin-6 and tumor necrosis factor-alpha levels. Pre-incubation of the plasma samples with a polystyrene-divinylbenzene-based adsorbent (30 nm average pore size) reduced endothelial intercellular adhesion molecule-1 expression to baseline levels, resulting in significantly diminished monocyte adhesion. Our data support the potential of porous polystyrene-divinylbenzene-based adsorbents to reduce endothelial activation under septic conditions by depletion of a broad range of inflammatory mediators.

**Electronic supplementary material:**

The online version of this article (doi:10.1007/s10753-016-0408-1) contains supplementary material, which is available to authorized users.

## INTRODUCTION

As a barrier between the blood stream and the surrounding tissues, the endothelium orchestrates tissue homeostasis, angiogenesis, and hemostasis; contributes to innate immunity; and plays a major role in regulating the physiological as well as the pathological host response to infection [1, 2]. In systemic infection, activation of the endothelium by pathogen-associated molecular patterns or by host-derived mediators such as chemokines, cytokines, and complement factors induces a shift of the endothelial surface towards a pro-coagulant and adhesive state. Excessive recruitment and adhesion of immune cells trigger sustained endothelial activation, increased vascular permeability, and impaired microcirculation, which are centrally involved in a variety of inflammatory conditions including sepsis and sepsis-associated multiple organ failure [3, 4].

The development of adjunctive sepsis therapies remains a major challenge due to the complex pathogenesis of this inflammatory syndrome and due to the vast heterogeneity of septic patients [5–7]. Extracorporeal therapies, such as high-volume hemofiltration, high cut-off hemodialysis, coupled plasma filtration adsorption, and, in particular, hemosorption have delivered promising pre-clinical and early clinical results, but have not yet been translated into clinical routine [8–10]. A potential advantage of extracorporeal therapies is that they affect only excess circulating pools of inflammatory mediators and target a wide range of molecules, whereas the systemic administration of specific antagonists leads to a complete blockade of their targets also in tissues, which may actually be harmful under certain circumstances. In addition to the depletion of soluble mediators, extracorporeal therapies have been proposed to act at the cellular level, modulating immune function by direct or indirect interaction with immune cells [11, 12].

We have previously shown that adsorption of inflammatory mediators from lipopolysaccharide-stimulated whole blood reduces activation of human umbilical vein endothelial cells (HUVEC) in a static cell culture model [13, 14]. *In vivo*, however, the endothelium is exposed to shear forces inducing changes in morphology, cellular function, as well as gene expression [15], and physiological flow plays a major role in modulating the adhesion as well as the transendothelial migration of immune cells [16–18]. Here, we used a microfluidic approach to study endothelial activation induced by plasma from highly hypercytokinemic patients and demonstrate the ability of polystyrene-divinylbenzene-based adsorbents to reduce endothelial activation under septic conditions in a pore size-dependent manner.

## MATERIALS AND METHODS

### Chemicals and Reagents

Acid citrate dextrose solution A (ACD-A; 22.0 g/l trisodium citrate, 24.5 g/l glucose monohydrate, 7.3 g/l citric acid) was obtained from Fresenius Medical Care, Bad Homburg, Germany. Unfractionated heparin was purchased from Gilvasan Pharma, Vienna, Austria. LPS from *E.coli* (055:B5), medium 199 (M199), RPMI-1640, 4-(2-hydroxyethyl)-1-piperazineethanesulfonic acid (HEPES), ethylenediaminetetraacetic acid disodium salt (EDTA), penicillin-streptomycin, fetal bovine serum (FBS), human AB serum, phosphate buffered saline (PBS), sodium azide, calcium chloride dihydrate, and magnesium chloride hexahydrate were from Sigma-Aldrich, St. Louis, MO, USA. Dulbecco’s phosphate buffered saline (DPBS) without calcium and magnesium was purchased from Life Technologies, Paisley, UK. Endothelial cell growth supplement was from BD Biosciences, San Diego, CA, USA.

### Adsorbents

Amberchrom CG161C, Amberchrom CG300M (both from Dow Chemical, MI, USA), and CytoSorb (CytoSorbents Corporation, NJ, USA) were used in this study. All adsorbents are based on polystyrene-divinylbenzene copolymers. CG161C and CG300M are uncoated, while CytoSorb is coated with polyvinylpyrrolidone [19]. Prior to use, all adsorbents were extensively washed with distilled water and isotonic saline and autoclaved at 120 °C for 30 min. They were characterized by scanning electron microscopy using a TM-1000 Tabletop Microscope (Hitachi, Tokyo, Japan) after washing with 96 vol% ethanol, drying for 12 h at 100 °C and sputter coating with gold (Q150R ES, Quorum Technologies). The specific surface area and the pore size distribution of the adsorbents were determined by recording nitrogen adsorption and desorption isotherms at 77 K (liquid nitrogen) using an ASAP 2010 surface area and porosimetry analyzer (Micromeritics Instrument Corporation, USA). Data were analyzed using the Brunauer, Emmett, and Teller (BET) method for specific surface area determination. The average pore size diameter d_avg_ was calculated using the total pore volume V_total_ according to Gurwitsch’s rule and the specific surface area A as: d_avg_ = 4 V_total_/A [20, 21].

### Monocyte Isolation

Human primary monocytes were isolated from leukocyte reduction chambers of the TrimaAccel® automated blood collection system (Version 5.0, Gambro BCT, Lund, Sweden). The chambers were provided by the Clinic for Blood Group Serology and Transfusion Medicine, Medical University of Vienna after approval of the study by the local ethics committee (ECS2177/2013). Monocyte isolation was performed as previously described [22, 23]. Purity and viability of monocytes were determined by flow cytometry after labeling with phycoerythrin (PE)-conjugated anti-CD14, pacific blue (PB)-conjugated anti-CD45 (Beckman Coulter, CA, USA), and fluorescein isothiocyanate (FITC)-conjugated annexin V (Becton Dickinson, NJ, USA) on a Gallios Flow Cytometer (Beckman Coulter, CA, USA) using the Kaluza software version 1.3 (Beckman Coulter, CA, USA).

### Cytokine Adsorption

Venous blood was freshly drawn from healthy volunteers after written informed consent and approval by the Institutional Review Board of Danube University Krems and anti-coagulated with ACD-A (1:40) and 0.8 IU/ml heparin. Blood was spiked with 100 ng/ml LPS and incubated for 4 h at 37 °C with gentle rolling. Stimulated blood was incubated with CG161C, CG300M, or CytoSorb for 1 h at 37 °C; plasma was collected by centrifugation (2000×*g*, 10 min, 4 °C) and stored at −80 °C until further use.

### Endothelial Activation Under Static Conditions

**Fig. 1:**
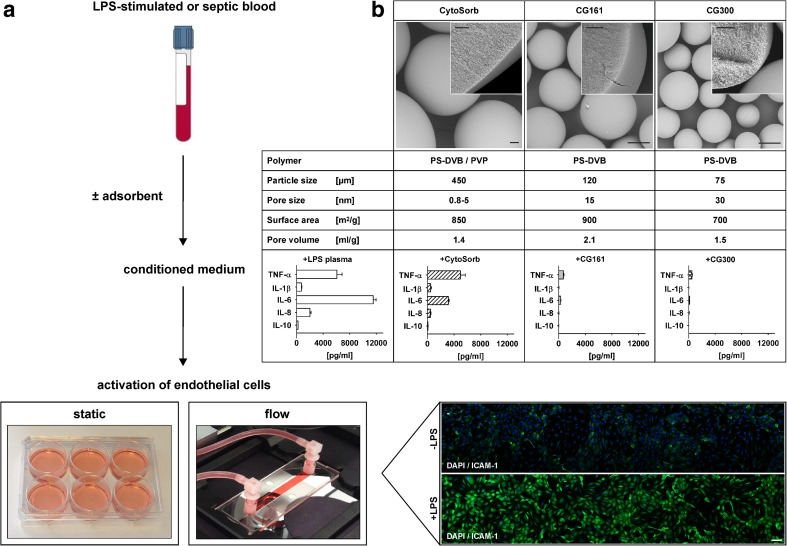
Cell culture model to study endothelial activation. Plasma from LPS-stimulated whole blood or from sepsis patients was diluted tenfold in cell culture medium to prepare conditioned medium, which was used to stimulate human umbilical vein endothelial cells under static conditions or under flow (**a**). The effect of cytokine depletion on endothelial activation was investigated by treatment of LPS-stimulated blood with the polystyrene-divinylbenzene (PS-DVB)-based adsorbents CytoSorb, CG161, and CG300 (**b**, *top*), which resulted in substantial reduction of TNF-α, IL-1β, IL-6, IL-8, and IL-10 (**b**, *bottom*). Data are given as mean ± SD, *n* = 3. *Scale bars* for the electron micrographs represent 50 μm (*overview*) and 10 μm (*insert*). *PVP* polyvinylpyrrolidone.

HUVEC were isolated as previously described [13] and seeded into 6-well plates at 3.5 × 10^5^ cells/well. Plas-ma obtained after treatment of LPS-stimulated whole blood with CG161C, CG300M, or CytoSorb or plasma from LPS-stimulated whole blood without adsorbent treatment (control) was supplemented with 60 IU/ml heparin, diluted tenfold in serum-free M199 to obtain conditioned medium, applied to HUVEC (1.5 ml per well), and incubated for 15 h in humidified atmosphere at 37 °C and 5 % CO_2_ (Fig. 1a). Supernatants were harvested, centrifuged at 500×*g* for 5 min at 4 °C, and stored at −80 °C until analysis. HUVEC surface expression of intercellular adhesion molecule (ICAM)-1 and E-selectin was analyzed by flow cytometry as described below. HUVEC viability was assessed using the EZ4U cell proliferation and cytotoxicity assay (Biomedica, Vienna, Austria).

### Endothelial Activation Under Flow

HUVEC were seeded into fibronectin-coated flow chamber slides (μ-Slide, Ibidi, Martinsried, Germany) at a density of 1 × 10^6^ cells/ml and incubated for 2 h under static conditions in humidified atmosphere (37 °C, 5 % CO_2_) to allow the cells to adhere. The slides were connected to the fluidic unit generating a unidirectional continuous flow, and HUVEC were adopted to flow at 2 dyn/cm^2^ for 1 h. Thereafter, the shear was increased to 5 dyn/cm^2^. At day 5, HUVEC were stimulated with conditioned medium (see above) for 4 h. THP-1 cells (American Type Culture Collection, Nr. TIB-202) or freshly isolated primary human monocytes suspended in serum-free cell culture medium (0.5 × 10^6^ cells/ml) were added to the reservoirs of the fluidic unit and perfused over HUVEC at 1 dyn/cm^2^ for 15 min. Adhering monocytes were visualized with an inverted Axiovert 40 CFL microscope (Carl Zeiss, Oberkochen, Germany) and quantified using ImageJ (NIH, USA). Expression of ICAM-1 and E-selectin was analyzed by immunofluorescence as described below.

### Activation of Endothelial Cells by Septic Plasma and Effect of Mediator Adsorption

Plasma samples from sepsis patients with high cytokine levels were tested for their potential to induce endothelial activation in the microfluidic model. Six samples from two patients were obtained at admission to the intensive care unit (0 h) and after 1 h and 24 h, respectively. The samples were provided by the University Clinic St. Poelten, Austria, after approval by the local ethics committee (GS4-EK-3/082-2012). Plasma samples were incubated for 1 h with 10 vol% of CG300M for cytokine adsorption or left untreated. After removal of the adsorbent by centrifugation and addition of 60 IU/ml heparin, the samples were diluted tenfold in serum-free medium M199 to obtain conditioned medium, which was used to stimulate HUVEC for 4 h at 5 dyn/cm^2^. Monocyte adhesion was quantified as described above.

### Quantification of Mediators

The Bio-Plex Pro^TM^ human cytokine 27-plex assay (Bio-Rad, Vienna, Austria) was used to quantify interleukin (IL)-1 beta, IL-2, IL-4, IL-5, IL-6, IL-7, IL-8, IL-9, IL-10, IL-12p70, IL-13, IL-15, IL-17A, and IL-1 receptor antagonist (IL-1ra); basic fibroblast growth factor (bFGF); granulocyte colony-stimulating factor (G-CSF); granulocyte-macrophage colony-stimulating factor (GM-CSF); interferon-gamma (IFN-γ); the chemokines CXCL10 (IP-10, interferon-inducible protein 10), CCL2 (MCP-1, monocyte chemotactic protein-1), CCL3 and CCL4 (MIP-1α and MIP-1β, macrophage inflammatory protein-1 alpha and beta); CCL5 (RANTES, regulated on activation, normal T-cell expressed and secreted); CCL11 (eotaxin, eosinophil chemotactic protein); platelet-derived growth factor (PDGF); tumor necrosis factor-alpha (TNF-α); as well as vascular endothelial growth factor (VEGF). Selected plasma samples were further characterized with a membrane-based antibody array (Proteome Profiler^TM^ ARY002, R&D Systems, Minneapolis, USA) detecting 102 cytokines, chemokines, and growth factors. Plasminogen activator inhibitor-1 (PAI-1) and human endothelial cell-specific molecule-1 (ESM-1, endocan) were quantified by enzyme-linked immunosorbent assays (Zymutest, Hyphen BioMed, Neuville-sur-Oise, France, and Lunginnov, Lille, France, respectively).

### Flow Cytometry

Endothelial activation was assessed by ICAM-1 and E-selectin expression. After stimulation, HUVEC were detached from the wells with 0.02 % EDTA, washed with ice-cold PBS, stained with PE-conjugated anti-CD62E and PE-Cy5-conjugated anti-CD54 monoclonal antibodies (Becton Dickinson, NJ, USA), and analyzed by flow cytometry (FC500, Beckman Coulter, CA, USA). Data were analyzed using the FlowJo software version 7.6.5 (Tree Star Inc., CA, USA).

### Immunofluorescence

Cells were fixed with 4 % paraformaldehyde for 10 min, washed with PBS, and stained with PE-conjugated anti-CD54 (eBioscience, Vienna, Austria) and allophycocyanin (APC)-conjugated anti-CD62E (abcam, Cambridge, UK) monoclonal antibodies. Nuclei were stained with DAPI (4′,6-diamidino-2-phenylindole, Sigma-Aldrich, St. Louis, MO, USA) at a concentration of 1 μg/ml. Fluorescence images were acquired with a confocal laser scanning microscope LSM780 (Carl Zeiss, Oberkochen, Germany) using a 20× objective (numerical aperture 0.8) or a 10× objective (numerical aperture 0.3).

### Statistical Analysis

Statistical analysis was performed with SPSS version 21 (SPSS Inc., Chicago, IL, USA). For the comparison of two groups, the nonparametric Wilcoxon rank sum test was applied and significance was accepted at *P* ≤ 0.05. Data are expressed as mean ± standard deviation (SD) or standard error of the mean (SEM) as indicated in the figure legends.

## RESULTS

### Adsorbent Characteristics

The physico-chemical characteristics of the polystyrene-divinylbenzene based adsorbents CytoSorb, CG161, and CG300 which were used for cytokine depletion in this study are summarized in Fig. 1b. Average bead sizes were 450 μm for CytoSorb, 120 μm for CG161, and 75 μm for CG300, respectively; average pore sizes were 0.8–5, 15, and 30 nm for CytoSorb, CG161, and CG300, respectively; and total pore volumes were 1.4 ml/g for CytoSorb, 2.1 ml/g for CG161, and 1.5 ml/g for CG300.

### Adsorption of Inflammatory Mediators from LPS-Stimulated Blood Reduces Endothelial Activation Under Static Conditions

Treatment of LPS-stimulated whole blood with CG161 and CG300 reduced TNF-α, IL-1β, IL-6, IL-8, and IL-10 to less than 5 % of the initial concentration, while CytoSorb was less efficient in reducing the larger cytokines TNF-α and IL-6 as shown in Fig. 1b. Cytokine depletion was confirmed by 27-plex bead array analysis as summarized in Supplementary Table 1.

**Fig. 2:**
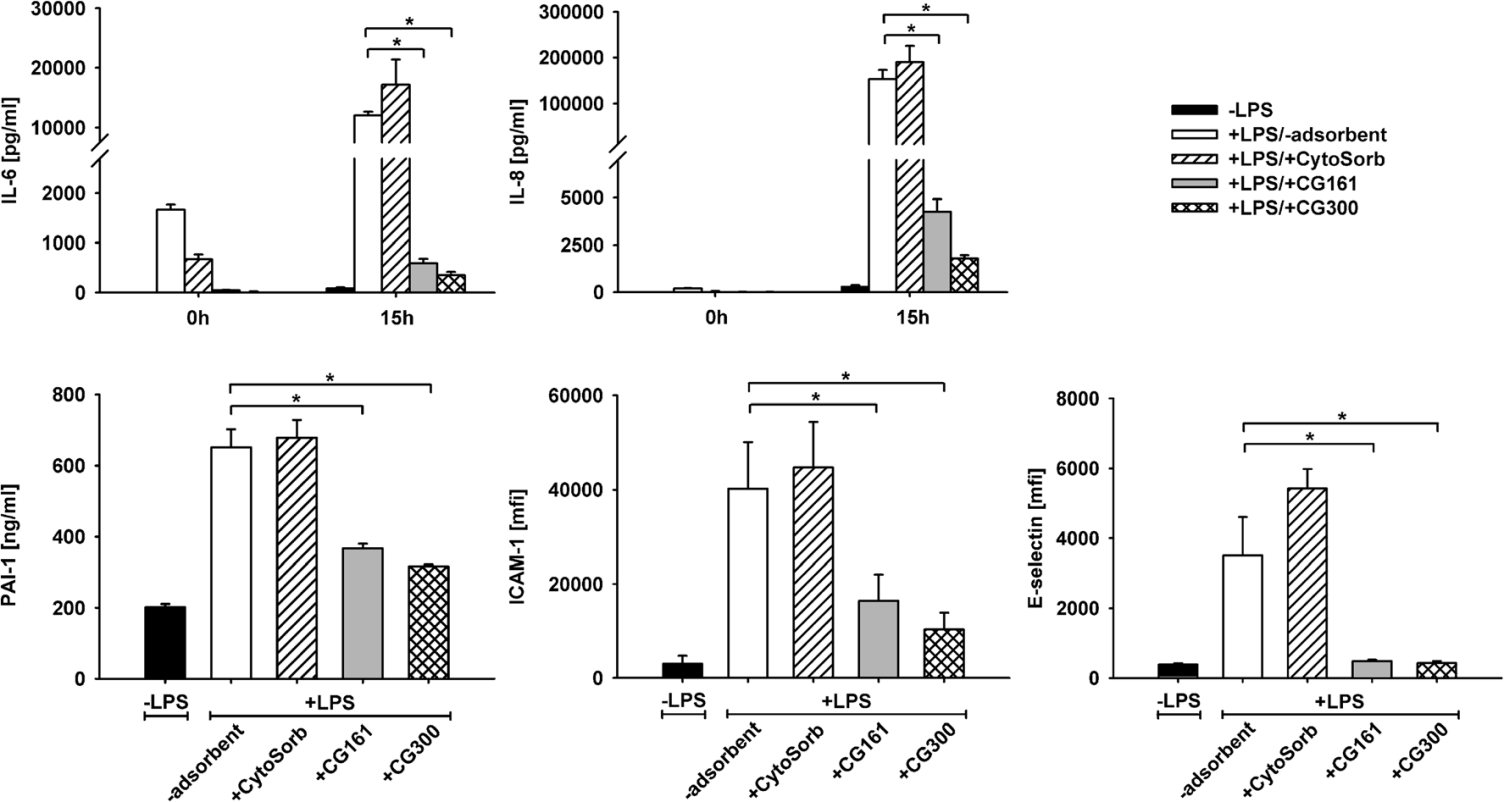
Effect of cytokine adsorption on endothelial activation under static conditions. Conditioned medium from adsorbent-treated or untreated lipopolysaccharide-stimulated whole blood was used to stimulate human umbilical vein endothelial cells. Endothelial activation was quantified after 15 h *via* IL-6 and IL-8 release, PAI-1 generation, as well as HUVEC surface expression of ICAM-1 and E-selectin. Time point 0 h refers to the cytokine levels in the conditioned media at the onset of the stimulation experiments. Data are given as mean ± SD for three independent experiments. *mfi* mean fluorescence intensity. Statistically significant differences (*p* < 0.05) are marked by asterisks.

Conditioned medium from LPS-stimulated whole blood induced HUVEC activation, as evidenced by increased IL-6 and IL-8 release, increased PAI-1 secretion, and by significant upregulation of ICAM-1 and E-selectin (Fig. 2). Treatment of LPS-stimulated blood with CG161 or CG300 prior to the preparation of conditioned medium resulted in strongly reduced endothelial release of IL-6 and IL-8, diminished secretion of PAI-1, as well as reduced expression of ICAM-1 and E-selectin as compared to the control without adsorbent. Pre-treatment of stimulated blood with CytoSorb failed to reduce endothelial activation. HUVEC viability was close to 100 % in all experiments.

### Adsorption of Inflammatory Mediators Reduces Monocyte Adhesion Under Flow

**Fig. 3:**
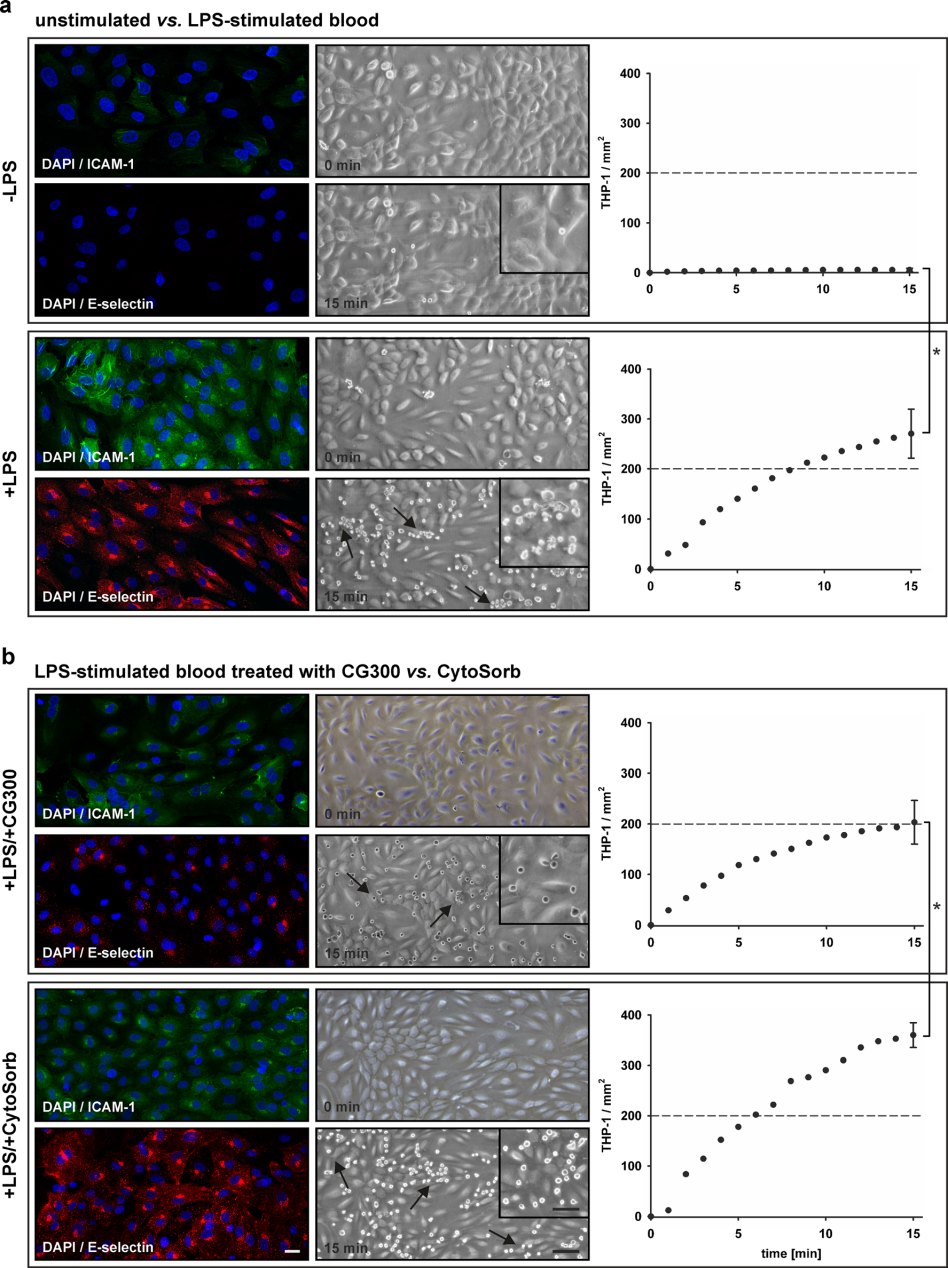
Activation of endothelial cells under flow. Human umbilical vein endothelial cells were treated with conditioned medium from unstimulated whole blood *vs.* lipopolysaccharide-stimulated whole blood (**a**) for 4 h at 5 dyn/cm^2^. Endothelial activation was assessed by surface expression of ICAM-1 and E-selectin and by adhesion of monocytic THP-1 cells. Treatment of LPS-stimulated blood with CG300 reduced endothelial activation as compared to treatment with CytoSorb (**b**). Data are expressed as mean ± SEM for three independent experiments. *Scale bars* represent 20 μm (immunofluorescence), 100 μm (light microscopy, *overview*), and 50 μm (light microscopy, *insert*).

Incubation of HUVEC with conditioned medium from LPS-stimulated blood resulted in enhanced ICAM-1 and E-selectin expression (mean fluorescence intensity 47.1 and 20.0 for ICAM-1 and E-selectin *vs.* 10.0 and 1.8 for the control) and in increased adhesion of monocytic THP-1 cells (270 ± 49 THP-1/mm^2^*vs.* 5 ± 3 THP-1/mm^2^ for the control; Fig. 3a), which was confirmed with primary human monocytes (Supplementary Fig. 1). Pre-treatment of LPS-stimulated blood with CG300 reduced ICAM-1 and E-selectin expression as compared to pre-treatment with CytoSorb (mean fluorescence intensity 18.8 and 10.0 for CG300 *vs.* 34.0 and 31.24 for CytoSorb), resulting in lower monocyte adhesion over time (Fig. 3b).

### Plasma Samples from Sepsis Patients Differ Regarding Endothelial Activation and Induction of Monocyte Adhesion

**Fig. 4:**
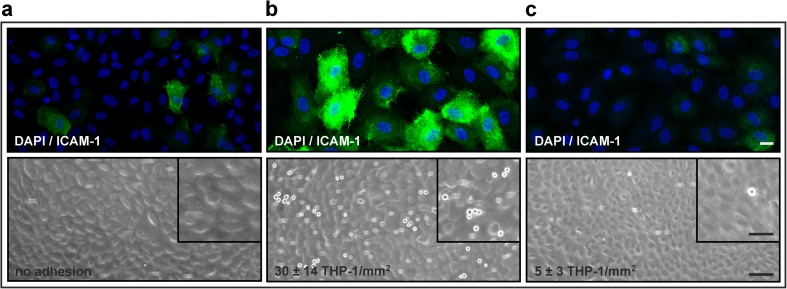
Adsorption of mediators from septic plasma reduces endothelial activation and monocyte adhesion. Plasma was obtained from sepsis patients at admission to the intensive care unit (0 h) and after 1 and 24 h. Representative results with plasma from two patients shown in **a** (patient 1) and **b** (patient 2) demonstrate the variable potential of septic plasma to induce endothelial activation. Both patients had comparable levels of IL-6 (>10,000 pg/ml). Pre-treatment of plasma from patient 2 with the adsorbent CG300 reduced ICAM-1 expression and monocyte adhesion to baseline levels (**c**). *Scale bars* represent 20 μm (immunofluorescence), 100 μm (light microscopy, *overview*), and 50 μm (light microscopy, *insert*).

In addition to conditioned medium from LPS-stimulated whole blood, we used plasma from sepsis patients to study the effect of cytokine adsorption on endothelial activation as described in “Materials and Methods.” For all plasma samples, IL-6 levels were >10,000 pg/ml at admission to the ICU (0 h) and at 1 h and >800 pg/ml at 24 h. TNF-α levels were 150 pg/ml at 0 h and 1 h and 20 pg/ml after 24 h. Despite these equal IL-6 and TNF-α levels, all samples from the time course of patient 1 failed to induce ICAM-1 upregulation and monocyte adhesion, while samples from series 2 triggered endothelial ICAM-1 expression and monocyte adhesion at all three time points (Fig. 4a, b). Pre-treatment of the plasma samples with CG300 reduced ICAM-1 expression and monocyte adhesion to baseline levels (Fig. 4c). According to cytokine bead array analysis, IL-1ra, IL-10, and IP-10 were significantly elevated in plasma samples from series 1 as compared to series 2 at all time points, while G-CSF and MCP-1 were decreased in samples from series 1 (Supplementary Fig. 2a and 2b). Analysis with a 102-plex membrane-based antibody array confirmed these findings. (Supplementary Fig. 2c and 2d).

### Differences in Plasma Levels of Endothelial Cell-Specific Molecule-1 Correlate With Differences in Monocyte Adhesion

To investigate the different potential of septic plasma to induce monocyte adhesion to the endothelium, we quantified ESM-1, which has previously been suggested to interfere with the binding of the major monocyte integrin LFA-1, to endothelial ICAM-1, resulting in reduced monocyte adhesion. Elevated ESM-1 levels were found in all samples from series 1 at all time points (0 h: 32.9 ng/ml *vs.* 11.8 ng/ml; 1 h: 40.0 ng/ml *vs.* 13.6 ng/ml; 24 h: 10.3 ng/ml *vs.* 6.4 ng/ml), correlating with decreased monocyte adhesion under flow.

## DISCUSSION

Sepsis is a leading cause of death in intensive care units and is among the top ten causes of global mortality. As a clinical syndrome, it comprises a variety of concomitant, integrated, and antagonistic processes involving both exaggerated inflammation and immune suppression. Most of the damage inflicted on the septic host can be ascribed to the innate immune response to invading pathogens. The endothelium, in particular, is a critical target of this host response. Excessive endothelial activation may spill over into the circulation and become uncoupled from inhibitory feedback mechanisms, converting a local adaptive inflammatory response into a systemic reaction.

To study endothelial activation under septic conditions and to investigate the influence of mediator adsorption with polystyrene-divinylbenzene-based polymers on endothelial activation, we used a static model as well as a microfluidic approach based on human umbilical vein endothelial cells and primary human monocytes or monocytic THP-1 cells. We stimulated HUVEC for 4 h under flow with conditioned medium containing plasma from LPS-stimulated whole blood to reflect the complexity of a septic environment. Monocytes were chosen to study leukocyte adhesion, as they act as sentinels in the earliest stages of sepsis and guide neutrophils towards inflammatory foci [24, 25]. Conditioned medium from LPS-stimulated whole blood induced reproducible endothelial activation and monocyte adhesion with little inter-assay variability in the flow model. Plasma samples from sepsis patients, in contrast, induced variable levels of endothelial activation despite their comparable levels of IL-6 and TNF-α. No such variability, however, was observed within a given time series, *i.e.*, among plasma drawn from the same patient at different time points. Further characterization of the plasma samples showed pronounced differences in IL-1ra, IL-10, IP-10, MCP-1, and G-CSF levels at all time points. The anti-inflammatory cytokines IL-1ra and IL-10 as well as the chemokine IP-10 were elevated in plasma samples that failed to trigger monocyte adhesion, with an at least fivefold increase in IP-10 at all time points. IP-10 is secreted by neutrophils, monocytes, as well as endothelial cells in response to interferon-γ. It is a key regulator of immune cell trafficking in infection and high IP-10 plasma concentrations have been shown to correlate with the severity of sepsis [26, 27]. MCP-1 and G-CSF, on the other hand, were elevated in plasma samples promoting activation of the endothelium and monocyte adhesion. MCP-1 is pivotal in monocyte recruitment during infection and stimulates migration of inflammatory monocytes from the bone marrow into the circulation. Consistent with our findings, it has been reported to trigger monocyte adhesion to activated endothelial cells under flow [28, 29]. G-CSF is the principal granulopoietic growth factor regulating the maturation of neutrophil precursors and has been found to increase the sensitivity to LPS by upregulation of lipopolysaccharide binding protein. Our results are in line with recent data reporting a 25-fold increase of MCP-1 and a 32-fold increase of G-CSF in plasma of severe sepsis patients [30].

With respect to the variable potential of plasma samples from different sepsis patients to induce monocyte adhesion, we argued that human endothelial cell-specific molecule-1, a proteoglycan released from endothelial cells under the control of pro-inflammatory cytokines, might interfere with monocyte adhesion *via* its binding to LFA-1 (CD11a/CD18) on the monocyte surface and blockade of LFA-1 interaction with ICAM-1 [31]. Supporting this hypothesis, we found elevated ESM-1 levels in all plasma samples that failed to induce monocyte adhesion.

Based on the rationale that restoration of immune homeostasis is essential in sepsis, extracorporeal therapies, such as high-volume hemofiltration, high cut-off hemodialysis, and plasma or hemosorption with polystyrene-divinylbenzene copolymers have been implemented to modulate a broad spectrum of inflammatory mediators, but adsorbent-based therapies have not yet been translated into clinical routine. We tested polystyrene-divinylbenzene-based adsorbents of different porosities for their effect on endothelial activation under septic conditions. Treatment of LPS-stimulated whole blood with CG161 and CG300 resulted in efficient depletion of inflammatory mediators and in strongly reduced endothelial activation both under static conditions and under flow, while no such reduction was observed with CytoSorb. It is likely that the lower average pore size of CytoSorb (0.8–5 *vs.* 15 nm for CG161 and 30 nm for CG300) accounted for this effect, since the pore size determines the accessibility of the inner surface of adsorbent polymers for target molecules and influences their adsorption capacity, in particular, for mediators of higher molecular mass, such as TNF-α (51 kDa) or IL-6 (21 kDa). Consistently, adsorbents with average pore sizes of 15 or 30 nm were more efficient in reduction of inflammatory mediators as compared to beads with average pores size below 5 nm (Supplementary Table 1).

While our data support the concept of extracorporeal mediator modulation as supportive approach to reduce endothelial activation, any adjunctive sepsis therapy targeting inflammatory mediators will depend on a combination with efficient point-of-care diagnostic systems to select patients who are likely to benefit from treatment at a given time point. Taken together, our findings highlight the subtle balance between beneficial innate immune reaction and detrimental inflammatory response to infection, which has to be taken into account in any therapeutic strategy aiming at a modulation of the host response in sepsis, precluding monotherapy or strategies targeting single mediators.

## ELECTRONIC SUPPLEMENTARY MATERIAL

Below is the link to the electronic supplementary material.

ESM 1 (PDF 852 kb)
