# Supplementary material for: Polystyrene-Divinylbenzene-Based Adsorbents Reduce Endothelial Activation and Monocyte Adhesion Under Septic Conditions in a Pore Size-Dependent Manner
Source: Inflammation. 2016 Aug 8;39(5):1737–46. doi: 10.1007/s10753-016-0408-1 (PMC5023745; doi:10.1007/s10753-016-0408-1)
Supplement: Supplementary file 1 — (PDF 852 kb) [file 10753_2016_408_MOESM1_ESM.pdf]

Supplement Material

Polystyrene-Divinylbenzene Based Adsorbents Reduce Endothelial Activation and Monocyte Adhesion Under Septic Conditions in a Pore-Size Dependent Manner

Tanja Eichhorn,[1] Sabine Rauscher,[2] Caroline Hammer,[1,2] Marion Gröger,[2] Michael B. Fischer,[1,3*] Viktoria Weber[1*]

[1] *Christian Doppler Laboratory for Innovative Therapy Approaches in Sepsis, Department for Health Sciences and Biomedicine, Danube University Krems, Krems, Austria*

[2] *Core Facility Imaging, Medical University of Vienna, Vienna, Austria*

[3] *Department of Blood Group Serology and Transfusion Medicine, Medical University of Vienna, Vienna, Austria*

*These authors contributed equally.

Corresponding author:

Viktoria Weber, Christian Doppler Laboratory for Innovative Therapy Approaches in Sepsis, Danube University Krems, Dr.-Karl-Dorrek-Strasse 30, 3500 Krems, Austria. Tel.: ++43 2732 893 2601; Fax: ++43 2732 893 4600; e-mail: viktoria.weber@donau-uni.ac.at

Supplementary Table 1. Cytokine adsorption from stimulated whole blood. Freshly drawn whole blood was stimulated with lipopolysaccharide (LPS) for 4 h and treated with 10vol% of adsorbent (CytoSorb, CG161, CG300). Plasma was collected by centrifugation (2000xg, 10 min, 4°C), and cytokine concentrations were determined using the Bio-Plex Pro™ human cytokine 27-plex bead array.

		+LPS/-adsorbent	+LPS/+CytoSorb	+LPS/+CG161	+LPS/+CG300
**IL-1β**	[pg/ml]	743	490	33	19
**IL-1ra**		2300	905	285	257
**IL-2**		45	17	6	5
**IL-4**		17	10	6	6
**IL-5**		54	28	39	38
**IL-6**		11538	3133	292	126
**IL-7**		8	1	0	0
**IL-8**		2021	431	38	51
**IL-9**		99	53	19	20
**IL-10**		218	84	16	8
**IL-12p70**		42	29	3	6
**IL-13**		6	1	1	1
**IL-15**		62	14	0	0
**IL-17A**		230	150	52	58
**Eotaxin**		137	74	49	43
**Basic FGF**		172	132	78	72
**G-CSF**		325	254	136	95
**GM-CSF**		258	117	98	89
**IFN-γ**		427	229	130	117
**IP-10**		5575	1261	717	555
**MCP-1**		1103	139	51	39
**MIP-1α**		19988	379	14	18
**MIP-1β**		12843	3509	440	585
**PDGF**		1546	423	232	111
**RANTES**		17938	13814	15498	13779
**TNF-α**		6070	4951	719	489
**VEGF**		58	38	6	2

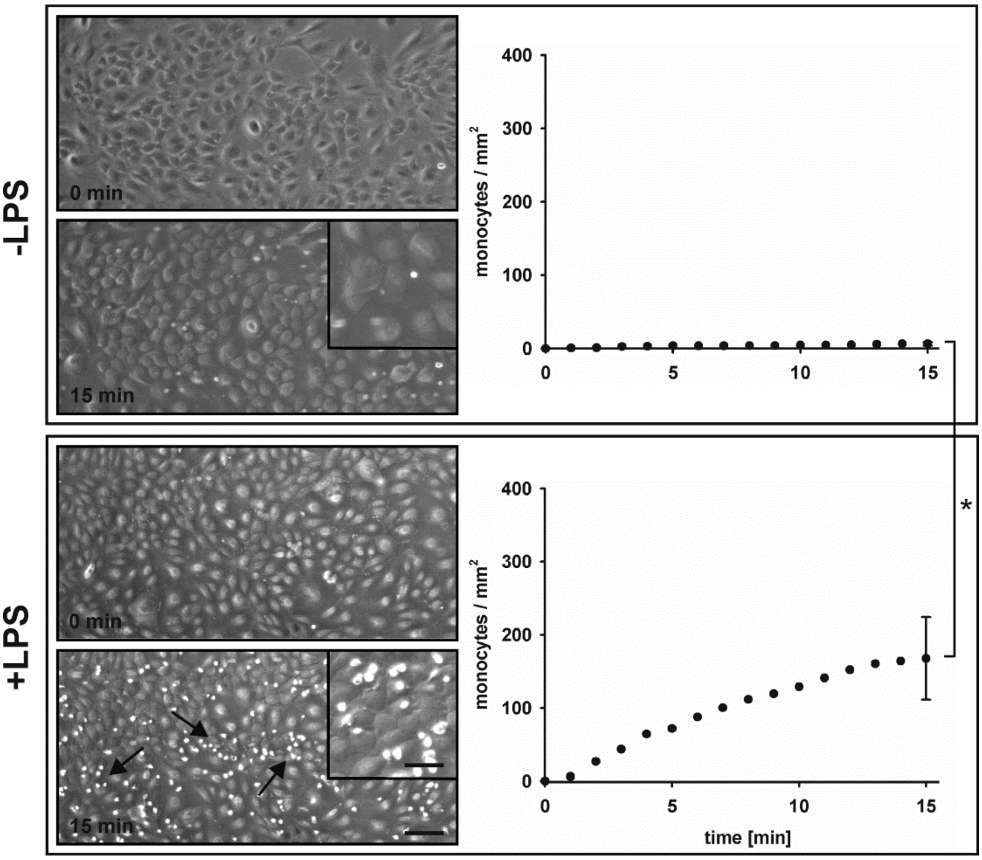

Supplementary Fig. 1. Adhesion of primary human monocytes to activated endothelium under flow. Human umbilical vein endothelial cells were incubated with conditioned medium from unstimulated and lipopolysaccharide-stimulated whole blood for 4 h at 5 dyn/cm^2. Endothelial activation was studied by circulation of freshly isolated primary human monocytes over the endothelial layer and quantification of monocyte adhesion over time. The scale bar represents 100 µm (overview) and 50 µm (insert). Data are expressed as mean ± SEM, n=3, * $P \leq 0.05$.

a

[pg/ml]	Patient 1 0h	Patient 1 1h	Patient 1 24h	Patient 2 0h	Patient 2 1h	Patient 2 24h
IL-1β	9	7	1	12	9	2
IL-1ra	3216	2149	184	719	708	151
IL-2	17	12	0	17	18	4
IL-4	9	8	1	9	7	2
IL-5	16	14	0	4	4	0
IL-6	11458	8583	789	10520	10810	743
IL-7	17	18	1	0	1	0
IL-8	262	212	66	446	319	119
IL-9	28	24	5	15	13	12
IL-10	75	73	11	35	35	19
IL-12p70	14	13	0	0	0	2
IL-13	5	4	1	2	0	0
IL-15	30	20	0	38	37	26
IL-17A	51	40	3	38	37	31
Eotaxin	77	61	18	62	63	18
Basic FGF	88	71	15	90	85	49
G-CSF	1115	935	370	7144	6641	608
GM-CSF	141	97	42	116	117	78
IFN-γ	241	204	29	180	166	16
IP-10	4723	4290	2877	576	597	585
MCP-1	170	145	60	553	517	436
MIP-1α	9	8	4	7	7	5
MIP-1β	172	158	105	147	139	275
PDGF	106	67	12	82	74	50
RANTES	944	762	335	1162	1166	841
TNF-α	150	123	17	137	141	24
VEGF	31	25	1	25	19	6

b

Patient 1 : Patient 2	0h	1h	24h
IL-1ra	4.47	3.04	1.22
IL-10	2.14	2.09	0.58
G-CSF	0.16	0.14	0.61
IP-10	8.20	7.19	4.92
MCP-1	0.31	0.28	0.14

c

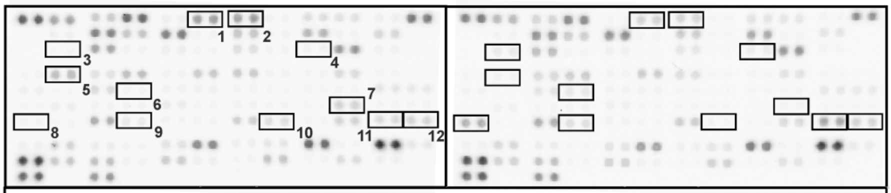

d

Patient 1 : Patient 2					
Angiopoetin-2	(1)	2.16	IP-10	(7)	6.92
BAFF	(2)	2.35	Leptin	(8)	0.07
ENA-78	(3)	0.28	MCP-1	(9)	0.54
G-CSF	(4)	0.57	MIG	(10)	4.78
Growth hormone	(5)	9.84	MIP-3β	(11)	0.37
IL-8	(6)	0.24	MMP-9	(12)	0.47

Supplementary Fig. 2. Characterization of plasma samples from sepsis patients. Two time series of plasma samples from sepsis patients with acute kidney failure were characterized with a 27-plex cytokine bead array. Samples were taken at admission to the intensive care unit (0 h), and after 1 h and 24 h. Cytokine concentrations were color coded using 10% increments with the lowest values shown in dark green and the highest values indicated in dark red (panel **a**). Five factors exhibited an at least two-fold difference between series 1 and 2, i. e., a ratio of >2 or <0.5 (panel **b**). Cytokine profiling at 0 h was complemented with a semi-quantitative membrane-based 102-plex antibody array (panels **c** and **d**). Numbers in brackets refer to the respective analyte spots of the 102-plex antibody array shown in panel **c**.
